# The Problem of Functional Paralysis

**Published:** 1914-07-25

**Authors:** 


### MEDICO-PSYCHOLOGY.

### The Problem of Functional Paralysis.

In the absence of any demonstrable evidence that those forms of paralysis which are conveniently termed functional are due to any lesion of the physical nervous system, neurologists are more than ever inclined to seek an explanation from the psychological side. Obviously, in view of the light which has been thrown on the pathogenesis of disorders of movement and sensation by the microscope in many nervous diseases, it was to be expected that a similar line of research would reveal some kind of change, possibly in the nerve-cell, if not in the nerve-fibre, which would account for the so-called "functional" group. Indeed, although the gross nervous changes in hysterical contractures or palsies cannot possibly be so definite as the degenerations which occur in disseminated sclerosis or myelitis, for example, nevertheless it is not improbable that opportunity might reveal some physical basis or accompaniment of the symptoms observed.

The difficulty, in fact, is, of course, that, while occasions not infrequently arise when examination may be made of the nervous system in cases of organic disease, it very rarely happens that the nervous tissues of hysterical persons come within the field of action of the morbid anatomist. Locomotor ataxy, spinal-cord tumours, intracranial syphilides, and similar conditions not uncommonly shorten life, while functional conditions very rarely lead to the *post-mortem* room. Thus, while investigations must be pushed in other directions at the present stage, it should never be forgotten that the microscope and scalpel may in the course of time help to elucidate the problems of hysteria, and make clear the physical basis which determines or is concomitant with the peculiar psychological conditions present.

### Vaso-motor Hypothesis.

Further, it must not be overlooked that a theory has already been put forward by Dr. Charlton Bastian—and was particularly supported by the late Dr. T. D. Savill—which does attempt in a reasonable manner to explain the physical basis of hysteria and the reason why, in the comparatively few cases that have been examined after death, the findings have been so unsatisfactory. This is the hypothesis that, just as in definite organic disease, functional paralysis is due to a real obstruction of the nerve-paths conducting the impulses from the central nervous system to the muscles, and whereas in the former injury, growth, or other gross lesion is readily demonstrable as the cause of the blockage, in the latter a similar condition may be the result of alterations in the blood-supply to various nerve-tracts; the consequent changes in the activities of nerve-cells and nerve-fibres preventing their proper functioning for the time being. Such a change in the blood-supply must be either of the nature of a flushing (hyperæmia) or emptying (anæmia) of various regions, and it must be supposed that this could only be brought about by a vaso-motor spasm or paralysis. If by any means it might be demonstrated that in functional paralysis there is a definite constriction or dilation of the blood-vessels supplying any particular part of the spinal cord or brain, then this vaso-motor theory would, indeed, be welcomed as the solution of a difficult problem. The supporters of the vaso-motor hypothesis admit the psychological aspects of hysteria, and, moreover, bring them to their aid by accepting it as a condition of their beliefs that it is an emotional stress that in the first place produces the abnormal vascular changes and subsequently maintains them. Unfortunately, this ingenious theory has not the support of any facts such as might reasonably be asked for by anyone who approaches the subject from the truly scientific standpoint.

### Psychology of Hysteria.

Attacking the subject as a problem of medico-psychology, the main fact that can be grasped is the proved similarity between hysterical phenomena and those produced by hypnotic suggestion, which leads readily enough to the hypothesis that the sufferer is the victim of his own perverted mentality, being quite unable to appreciate things as they are. To explain how any false idea can influence so complicated a faculty as the movement of a limb, it is necessary to suppose that every function becomes represented psychologically by corresponding systems of ideas—that is to say, of mental impres sions relating to sight, movement, and touch. Accepting this, it follows that, although an individual might have everything intact in his nerve-muscle mechanism, he might temporarily have complete loss of memory for the psychological complex (thought-group) representing the whole of a particular movement, and thus be unable to bring it about.

Certainly it is characteristic of hysterical persons to be unable to carry as many ideas in their minds as the normal individual, and it may be that in this temporary forgetfulness—which, be it noted, is not only in the ordinary field of conscious thought, but in the level of mentality which is nowadays commonly labelled sub-conscious—we have an explanation of functional paralysis. It is to Professor Janet, of Paris, that we owe the best exposition of this doctrine of amnesia as a main factor in hysteria.

Again, in some directions hysterics are very suggestible, and, this being so, it is quite possible that they will react, just as does the hypnotised subject, to a very slight suggestion in an abnormal degree. Thus some casual indirect suggestion may readily lead a hypnotic subject to lose the use of an arm for the time being, and it is quite possible that in a similar manner the subject of hysteria may have suggested to him, or even suggest to himself, the inability to move a limb. Such self-suggestion will be all the more likely to arise after the shock of an accident or emotional stress, and, indeed, it is not uncommon for functional paralysis to occur as the result of some condition of this kind. In support of the "suggestion" theory, which has been worked out most fully and with great persuasiveness by Professor Babinski, yet another Parisian neurologist, we have the undoubted fact that in regard to the disorders of sensation which are so characteristic of hysteria the distribution of anæsthesia very frequently may be traced to nothing more than suggestion or self-suggestion. Indeed, Babinski himself goes so far as to affirm that the anæsthesia observed in hysteria is invariably suggested by the methods of the examining physician. Be this as it may, it seems most likely that future progress will more likely be made in the study of hysteria by following the lines of research that Janet and Babinski have so ably advocated than any other. At the same time we must, of course, give credit to the painstaking researches of Professor Freud and his co-workers (whose theories have lately been outlined in The Hospital, May 16, 1914), although it does not now seem likely that their claims will be fully substantiated. Those who wish to follow up this fascinating subject will find an extensive critical summary of modern theories of hysteria in the reports of Dr. Ormerod's recent Lumleian Lectures delivered before the Royal College of Physicians.

